# Long-term treatment with subcutaneous immunoglobulin in patients with chronic inflammatory demyelinating polyradiculoneuropathy: a follow-up period up to 7 years

**DOI:** 10.1038/s41598-020-64699-6

**Published:** 2020-05-13

**Authors:** L. Gentile, A. Mazzeo, M. Russo, I. Arimatea, G. Vita, A. Toscano

**Affiliations:** Unit of Neurology and Neuromuscular Diseases, Department of Clinical and Experimental Medicine, University of Messina, Messina, Italy

**Keywords:** Neuroscience, Peripheral nervous system

## Abstract

Chronic Inflammatory Demyelinating Polyneuropathy (CIDP) is a rare and heterogeneous acquired sensory-motor polyneuropathy with autoimmune pathogenesis. Intravenous immunoglobulins (IVIG) are a well-established therapy for CIDP: it is well known that at least two-thirds of these patients need these infusions for several years. More recently, Subcutaneous Immunoglobulins (SCIg) have been proved to be effective: this finding has been confirmed either in isolated cases or in few randomized trials. However, it appeared that the longest SCIg treatment follow up lasted no longer than 48 months. We report herein the results of a long-term SCIg treatment with a follow up period up to 7 years (84 months), considering safety, tolerability and patients’ perception of SCIg treatment in a CIDP population. We studied 17 patients (10 M; 7 F) with a diagnosis of CIDP, defined according to the EFNS/PNS criteria, successfully treated with IVIG every 4/6 weeks before being switched to SCIg treatment. Clinical follow-up included, apart from a routinely clinical assessment, the administration of Medical Research Council (MRC) sum-score, the Overall Neuropathy Limitation Scale (ONLS) and the Life Quality Index questionnaire (LQI). The results showed that, in the majority of this pre-selected group of CIDP patients (16/17), SCIg were well tolerated and were preferred over IVIG. Strength and motor functions remained stable or even improved during the long term follow-up (up to 84 months) with benefits on walking capability and resistance, manual activity performances and fatigue reduction.

## Introduction

Chronic Inflammatory Demyelinating Polyneuropathy (CIDP) is a rare and heterogeneous acquired sensory-motor polyneuropathy with autoimmune pathogenesis. CIDP usually manifest with a progressive, relapsing–remitting or monophasic course and could lead patients to motor and/or sensitive impairment^1^. According to a recent systematic review, CIDP incidence is of 0.33 per 100.000 persons per year with a prevalence of 2.81 per 100.000^2^. The diagnosis of typical CIDP, or of its atypical variants, is based on a combination of clinical, electrodiagnostic and laboratory findings established by the European Federation of Neurological Societies/Peripheral Nerve Society (EFNS/PNS) task force in 2010^3,4^. Most of the CIDP patients become disable in motor daily life activities and their quality-of-life is sensibly decreased^1,5^. A timely and appropriate therapy start is often crucial to prevent permanent disability^6^. The primary goals of treatment are: decrease the clinical burden of CIDP, reduce sensory-motor symptoms, improve functional status (e.g., reduce disability and handicap) and maintain long-term remission as long as possible^7^. High dosage intravenous immunoglobulins (IVIG) are a well-established therapy for CIDP^8–10^: it is well known that at least two-thirds of these patients need infusions for several years^11^. More recently, subcutaneous immunoglobulins (SCIg) administration has been proved to be effective in CIDP patients responsive to IVIG as a maintenance treatment or, even, as a first line therapy^12–17^. However, from the literature data, it appears that the longest SCIg treatment follow up lasted no longer than 48 months^5,18,19^. We report herein the retrospective results of a long-term SCIg treatment with a follow up period up to 7 years (84 months), considering safety, tolerability, clinical outcome measures variations and patients’ perception of SCIg treatment in a CIDP population.

## Patients

We retrospectively examined 17 patients (10 M; 7 F), all >18 year-old, with a diagnosis of CIDP (see Table 1), defined according to the EFNS/PNS criteria, successfully treated with IVIG with a stabilization of their clinical conditions. All patients started IVIG administration every 4/6 weeks [IVIG mean duration: 3.3 years (0.5–11 yrs)] before switching to SCIg treatment. SCIg option was chosen because: (1) patients discomfort because the necessity of repeated and long journeys to the infusion site (16/17 pts.), (2) economical burden (9/17), (3) work problems when moving to the infusion site (10/17), (4) difficulties related to venous access (2/17 pts). A SCIg equivalent dose to IVIG has been used.

**Table 1:** Patients’ clinical characteristics, treatment data and outcome measures.

Pts	Age at last follow up (years)	Sex	Disease duration at last follow up	First line treatment (FLT)	FLT duration	IVIG duration	Dose SCIg (gr/week)	SCIg duration	ONLS		MRC s.s.		LQI	
									T0	T1	T0	T1	T0	T1
1	47	M	19 years	prednisone	12 years	1 year	20	6 years	2	2	78	78	66	90
2	77	M	14 years	prednisone	7 years	2 years	16	5 years	5	5	44	46	44	67
3	58	F	6 years	IVIG	4.5 years	4.5 years	30	5 years	5	5	59	62	59	81
4	54	F	12 years	predn/AZT	1 year	4 years	12	6 years (suspended)	4	4	64	64	85	86
5	48	M	12 years	AZT	4 years	4 years	20	7 years	3	2	66	66	75	99
6	79	M	7 years	IVIG	1 year	1 year	20	6 years	6	3	70	80	85	98
7	60	M	8 years	predn/AZT	1 year	0.5 year	18	7 years	2	1	68	80	82	90
8	74	F	7 years	IVIG	0.5 year	0.5 year	16	6 years	3	1	78	80	82	98
9	72	F	6 years	prednisone	1 year	0.5 year	12	5 years	7	6	69	73	62	78
10	60	F	17 years	prednisone	17 years (ongoing)	11 years	9.6	2 years (suspended)	4	6	68	60	62	70
11	68	M	6 years	prednisone	0.5 year	0.5 year	30	3.5 years	5	5	66	66	60	89
12	56	M	12 years	prednisone	12 years (ongoing)	2 years	30	4.5 years	2	2	75	75	63	92
13	27	M	12 years	IVIG	8 years	8 years	12.8	4 years	2	0	71	80	58	96
14	60	M	7 years	prednisone	0.5 year	0.5 year	15	3 years	2	0	78	80	75	100
15	64	F	13 years	predn/AZT	4 years	4 years	20	3 years	4	3	74	74	56	84
16	55	F	12 years	predn/AZT	0.5 year	4.5 years	12.8	7 years	3	3	76	76	78	95
17	47	M	14 years	prednisone	12 years (ongoing)	1 year	20	2 year	3	2	78	78	64	90

Pts: patients; ONLS: overall neuropathy limitation scale; MRC s.s.: medical research council sum score; LQI: life quality index questionnaire; T0: baseline (at SCIg treatment beginning); T1: last follow-up.

Among the CIDP patients, one patient was also affected by IgG lambda monoclonal gammopathy of undetermined significance (MGUS). Before SCIg, patients’ first line treatment (FLT) was IVIG only in 4/17 and prednisone/azathioprine in 13/17. Of these 13 patients, 10 (Pt. 1, 2, 4, 5, 7, 9, 11, 14, 15, 16) suspended the FLT because side effects (such as severe osteoporosis, high blood pressure level, glaucoma) and/or poor improvement. In this group, prednisone/azathioprine treatment mean duration was of 3.1 years. The remaining 3 patients continued steroids: Pts. 12 and 17 at low daily dose combined with SCIg, whereas pt. 10 suspended SCIg infusion and kept assuming only steroids. As regard as Pts. 12 and 17, it was not possible to stop steroids either during IVIG or during SCIg infusions.

## Methods

Before starting SCIg treatment at home, the patients were trained in the hospital with nurse assistance. Then, the treatment was self-administred at home via a programmable infusion pump (chrono-speed 50 by Canè S.p.a, Italy) coupled to a 50 mL syringe connected with catheters to a butterfly subcutaneous needle. All patients signed an informed consent form and the study has been approved by the Ethics Committee of Messina (address: AOU “G.Martino”, via Consolare Valeria n.1, 98125 – Messina (ME), Italy). This protocol has been performed in accordance with the ethical standards laid down in the 1964 Declaration of Helsinki and its later amendments. We considered baseline records and follow-up data collected between 2013 and 2020; during this period, patients were evaluated at baseline and every 6-months.

Clinical follow-up included:

1. Medical Research Council (MRC) sum-score to check muscle strength (0 = complete paralysis, 80 = normal strength) bilaterally in eight muscle groups (shoulder abduction, elbow flexion, wrist extension, index finger abduction, hip flexion, knee extension, foot dorsiflexion and great toe dorsiflexion).
2. The Overall Neuropathy Limitation Scale (ONLS: 0 = normal, 11 = worst) to assess motor disability.
3. The Life Quality Index questionnaire (LQ I) as a quality of life measures. 15-items examining the respondent’s perceptions of immunoglobulin treatment impact on daily activities, summarized to four sub-scales: “treatment interference”, “therapy related problems”, “therapy setting” and “treatment costs”.

Patients with ONLS reduction of at least 1 point were considered improved. Neither MRC score nor ONLS scale variations were considered as evidence of strength or motor ability stabilization. Relapses were identified as clinical deterioration with increase of ONLS and decrease of MRC sum-score at least of one point. In case of relapse, SCIg dose was increased by 20% for 5 weeks. If the increase did not produce clinical improvement, a IVIG course (at pre-relapse dose) was administered to the patient during SCIg treatment. These patients were then revaluated after 2 weeks: if the clinical condition improved, IVIG course was not repeated and SCIg treatment only was continued.

## Results

Patients median age was of 59 years (27–79 yrs.), with mean disease duration of 11 years (6–19 yrs.) (Table 1). SCIg mean duration was 4.8 years (2–7 yrs.) and their average dose was of 18.5 g/week. Almost all patients (15/17) showed a good SCIg compliance except for two (pts 4 and 10) out of 17. These 2 patients, after respectively 6 and 2 years of SCIg course, decided to stop the treatment. Pt 4 spontaneously decided to return to IVIG for personal reasons, despite a clear clinical stabilization. Patient 10, affected by IgG lambda MGUS, with a slow progressive course and an initial mild response to IVIG and steroids, was not particularly satisfied of SCIg; she continued with steroids and IVIG boluses, still with mild benefit. Interestingly, when submitted to LQI score, all 17 patients evidenced an improvement. Moreover, in 16 out of 17 patients, ONLS score and MRC sum score improved (8/17) or stabilized (8/17). During the follow up, two patients (Pt. 2 and 13) relapsed, respectively 4 and 36 months after SCIg start. Although SCIg dosage was increased of 20%, no improvement was shown after 5 weeks. Administration of a single IVIG course, associated to SCIg, made a clinical improvement after 2 weeks with no further necessity of IVIG. Finally, We observed no serious adverse events; an occasional mild/moderate skin reaction at infusion sites was reported in 6/17 pts.

## Discussion

CIDP is a rare chronic disease, highly disabling. In randomized clinical trials, steroids, plasma-exchange and IVIG have been shown similar efficacy, with approximately a 50–70% responder rates for each treatment^10,20^. This has been confirmed by at least two Cochrane reviews and it has also been reported on the “Treatment Guidelines of the European Federation of Neurological Societies/Peripheral Nerve Society”^21–23^. Currently, IVIG are used as the first therapeutic intervention for CIDP, given less frequent side effects and frequent short-term efficacy than steroids^10,20^. In several small trials, IVIG treatment was suggested to be efficacious versus plasma exchange, prednisolone and placebo^24^, with two-thirds of patients with CIDP needing long-term intravenous immunoglobulin treatment^12^.

In the last years, many studies have demonstrated SCIg administration effectiveness. A randomized, double blind, placebo controlled study on CIDP patients, initially treated with IVIG and, then, switched to placebo or SCIg, has demonstrated an improvement of muscle strength in patients treated by SCIg compared to the placebo group^13^. A multicenter, prospective study of 66 CIDP patients on IVIG therapy and switched from monthly IVIG to weekly SCIg, showed an improvement of the overall neuropathy limitation score (ONLS) and a stabilization of MRC sum score^14^. Another randomized, controlled, cross-over study showed the effect of SCIg in 2 groups of “de novo” patients: one treated with a single dose IVIG and the second by SCIg for 5 weeks. A similar muscle strength improvement was observed in both groups^12^. A multicenter controlled trial in 172 patients, who relapsed after IVIG withdrawal, demonstrated the efficacy as maintenance treatment of either low dose or high dose SCIg treatment versus placebo, ending with an absolute risk relapses reduction of 25% with a low dose and 30% with a higher dose^15,18^. These results were confirmed by an open-label prospective extension study, which showed that SCIg treatment provided long-term benefit either by 0.2 or 0.4 g/kg weekly dose with a lower relapse rates with the higher dose^16^. Recently, long-term SCIg treatment have been demonstrated to be effective in improving and/or maintaining strength, sensitivity, motor ability on daily life activities and quality of life since 24 to 48 months after treatment start. In this study, some neurophysiological parameters (distal compound motor action potential and sensory nerve action potential) were reported as useful prognostic factors^17^. Among the above mentioned trials, it appeared that the longest SCIg treatment follow up went on no longer than 48 months^5,18,19^.

Our study showed that, in most of this pre-selected group of CIDP patients (16/17), SCIg were well tolerated and preferred by patient over IVIG. Strength and motor functions remained stable or, even, improved during long term follow-up (up to 84 months) with benefits on walking capability and resistance, hand fine activities and fatigue reduction. Relapse rate was of 23% (4/17): during follow-up, two patients presented episodic clinical worsening and were successfully treated with occasional IVIG cycles. Only one patient with IgG lambda MGUS comorbidity, did not respond to SCIg, which were interrupted. SCIg administration was usually well tolerated; some patients reported only minor and rapidly reversible adverse events. A global personal satisfaction was declared by patients during SCIg treatment as a significant improvement of their quality of life. Interestingly, when compared to IVIG treatment, patients appreciated the possibility of injecting themselves at home. They also positively evaluated the possibility to continue to work or spend their social and daily life activities without the continuous necessity to afford any extra-cost to reach the infusion centre and/or to reside in proximity of it because of the repeated IVIG infusion.

A limitation of this study could be considered being a retrospective study with a relatively small sample size of patients. Two patients were on double treatment with SCIg and steroids, and this might have impacted on the global results. Prolonged observation of larger group of CIDP patients, not undergoing any other therapy and associated with tapering or cessation of treatment, would be necessary to better assess the effects of long-term SCIg treatment.

The main strength of our study is the long-term follow up (up to 7 years), that strongly confirm safety and tolerability of SCIg. Patients perception of treatment is very positive, with SCIg clearly preferred to IVIG as a chronic treatment. This results strengthen the recommendation to use SCIg as a very good choice as chronic therapy in CIDP patient IVIG responders.
